# Enhancement of mechanical and corrosion resistance properties of electrodeposited Ni–P–TiC composite coatings

**DOI:** 10.1038/s41598-021-84716-6

**Published:** 2021-03-05

**Authors:** Osama Fayyaz, Adnan Khan, R. A. Shakoor, Anwarul Hasan, Moinuddin M. Yusuf, M. F. Montemor, Shahid Rasul, Kashif Khan, M. R. I. Faruque, Paul C. Okonkwo

**Affiliations:** 1 Center for Advanced Materials (CAM), Qatar University, 2713 Doha, Qatar; 2 Department of Mechanical and Industrial Engineering, College of Engineering, Qatar University, 2713 Doha, Qatar; 3 Departamento de Engenharia Química, Centro de Química Estrutural, Instituto Superior Técnico, Universidade de Lisboa, Av Rovisco Pais, 1049-001 Lisboa, Portugal; 4 Department of Mechanical and Construction Engineering, Northumbria University, Newcastle, UK; 5 School of Mechanical, Aerospace and Automotive Engineering, Coventry University, Coventry, UK; 6 Space Science Centre, Institute of Climate Change of the Universiti Kebangsaan Malaysia (UKM), Bangi, Malaysia; 7 Department of Mechanical and Mechatronics Engineering, College of Engineering, Dhofar University, Salalah, Oman

**Keywords:** Nanoscale materials, Structural materials, Materials science

## Abstract

In the present study, the effect of concentration of titanium carbide (TiC) particles on the structural, mechanical, and electrochemical properties of Ni–P composite coatings was investigated. Various amounts of TiC particles (0, 0.5, 1.0, 1.5, and 2.0 g L^−1^) were co-electrodeposited in the Ni–P matrix under optimized conditions and then characterized by employing various techniques. The structural analysis of prepared coatings indicates uniform, compact, and nodular structured coatings without any noticeable defects. Vickers microhardness and nanoindentation results demonstrate the increase in the hardness with an increasing amount of TiC particles attaining its terminal value (593HV_100_) at the concentration of 1.5 g L^−1^. Further increase in the concentration of TiC particles results in a decrease in hardness, which can be ascribed to their accumulation in the Ni–P matrix. The electrochemical results indicate the improvement in corrosion protection efficiency of coatings with an increasing amount of TiC particles reaching to ~ 92% at 2.0 g L^−1^, which can be ascribed to a reduction in the active area of the Ni–P matrix by the presence of inactive ceramic particles. The favorable structural, mechanical, and corrosion protection characteristics of Ni–P–TiC composite coatings suggest their potential applications in many industrial applications.

## Introduction

Corrosion is the gradual destruction of metal because of the chemical reaction with its environment. Corrosion has a large share in the failure of equipment and loss of production. Corrosion behaves like a slow poison for the destruction of industrial finished products, machinery, pipelines from onshore to offshore sites etc^1,2^. Corrosion is the major challenge faced by many industries nowadays due to various failures such as fatigue stress initiation and creep failure rooting back to corrosion^3^. Corrosion of valves in the reverse osmosis system results in equipment failure^4^. The loss of containment in the onshore pipelines is threatened by the corrosive environment^5^. One of the various corrosion types, such as pitting, is one of the hazardous forms of corrosion in marine and offshore structures^6^. Nearly 10 to 30% of the maintenance budget is spent on corrosion control by the oil refinery plants, as deduced by Finšgar et al.^7^. Shekari et al.^8^ mentioned the report of NACE, which estimated the global cost of corrosion to be US$2.5 trillion in 2013, which was equivalent to 3.4% of the Gross Domestic Product (GDP).

Understanding of corrosion mechanism has led to the development of various techniques to prevent and minimize corrosion damages. Surface modification techniques provide a dual benefit of corrosion prevention and improvement of the surface properties such as hardness, abrasion, wear, inertness, and erosion, avoiding replacing the bulk of material^9^. Various surface modification techniques like carburizing, nitriding, carbonitriding, flame hardening, laser hardening, chemical vapor deposition and physical vapor deposition, etc. have been reported in the literature^10^. Providing a barrier between the corroding environment and the base metal with a corrosion-resistant layer is termed as a coating, which is primarily applied to prevent the loss of metal. The coating of base metal with a varying thickness can be carried out in various ways^11^. Electrodeposition coating has gained wide acceptance in academia and industries due to its cost-effectiveness, simplicity, and capability to produce expeditious results^12,13^. It is also used in the decorative sector, and the growth of the electroplating market is forecasted to reach US$ 21 billion by 2026^14^.

Ni–P coatings have found applications in numerous industries such as aerospace, electronics, and automotive due to their good wear resistance, a higher degree of hardness, lower friction coefficient, and interesting anti-corrosive resistance^15^. A careful selection of coating bath composition and optimization of electrodeposition parameters is vital to achieving the desired properties of Ni–P coating, leading to widening their range of applications^16,17^. There are mainly two proposed mechanisms for the formation of Ni–P coatings over a substrate in the respective chemical bath and operating conditions, namely direct and indirect mechanisms. Among these two, the latter i. e. indirect coating mechanism is mainly supported by the majority of the researchers. More details about the mechanism of electrodeposition of Ni–P coatings on the substrates can be glanced in the review^16^. Ni–P coatings have the edge over other alloy coatings such as Ni–Cu, Ni–Fe, and Ni–Co and even Ni-composites for the fabrication of microsystems^18^. For instance, Ni–P–Co coatings are reported to have better hardness and lubricity, along with many other appealing characteristics^19,20^. Various chemical baths consisting of sulfate, sulfamate, and methanosulfonate have been reported in the literature for obtaining Ni–P coatings^21^.

Co-deposition of reinforcing particles to enhance Ni–P coatings specific properties through composites formation is a leading trend in the academic and classical industries^22–24^. Recently, research in the area of Ni–P composite coatings is quite common, which has led to the development of some novel composite coating systems^9,25–33^. Although the Ni–P–X (X = TiO_2_, SiO_2_, ZrO_2_, CeO_2_ etc.) composite coatings are grabbing substantial attention^34–37^, the effect of electrodeposited titanium carbide (TiC) has not been fully investigated in spite of its attractive properties such as high hardness, wear resistance, corrosion resistance and high stability at elevated temperature^38,39^. The present study deals with the synthesis and characterization of Ni–P–TiC composite coatings developed through conventional electrodeposition techniques. This work is mainly focused on the electrodeposition which is completely different technique from electroless deposition. Also, the chemical bath modified and the optimized parameters for our study is completely different from the previously reported work. Moreover, our study also considers the effect of increasing the TiC particles (< 200 nm) which on one hand improves the mechanical properties through matrix-reinforcement composite phenomenon and on other hand improves the corrosion resistance by blocking the active surface area. This further endorses the novelty of our present study that the effect of various TiC particles concentrations on the structural, surface, mechanical, and corrosion-resistant properties of Ni–P coatings have been deeply investigated. The results evidence an improvement in the mechanical properties and corrosion resistance supporting the use of Ni–P–TiC composite coatings for onshore and off shore pipelines^40^, tool finish and machining hard surfaces^41^, microsystems and micro engines^18^, as a replacement for hard chromium coatings^16^, and catalytic coatings for hydrogen evolution in water electrolysis^16^ etc.

## Material and methods

### Materials

Nickel sulphate hexahydrate, nickel chloride hexahydrate, boric acid, orthophosphoric acid, and sodium hypophosphite were bought from the Sigma Aldrich, Germany. Sodium chloride and submicron-sized titanium carbide (TiC) powder with an average particle size < 200 nm and purity of 99.9% were also imported from Sigma Aldrich.

### Sample preparation and coatings synthesis

**Figure 1:**
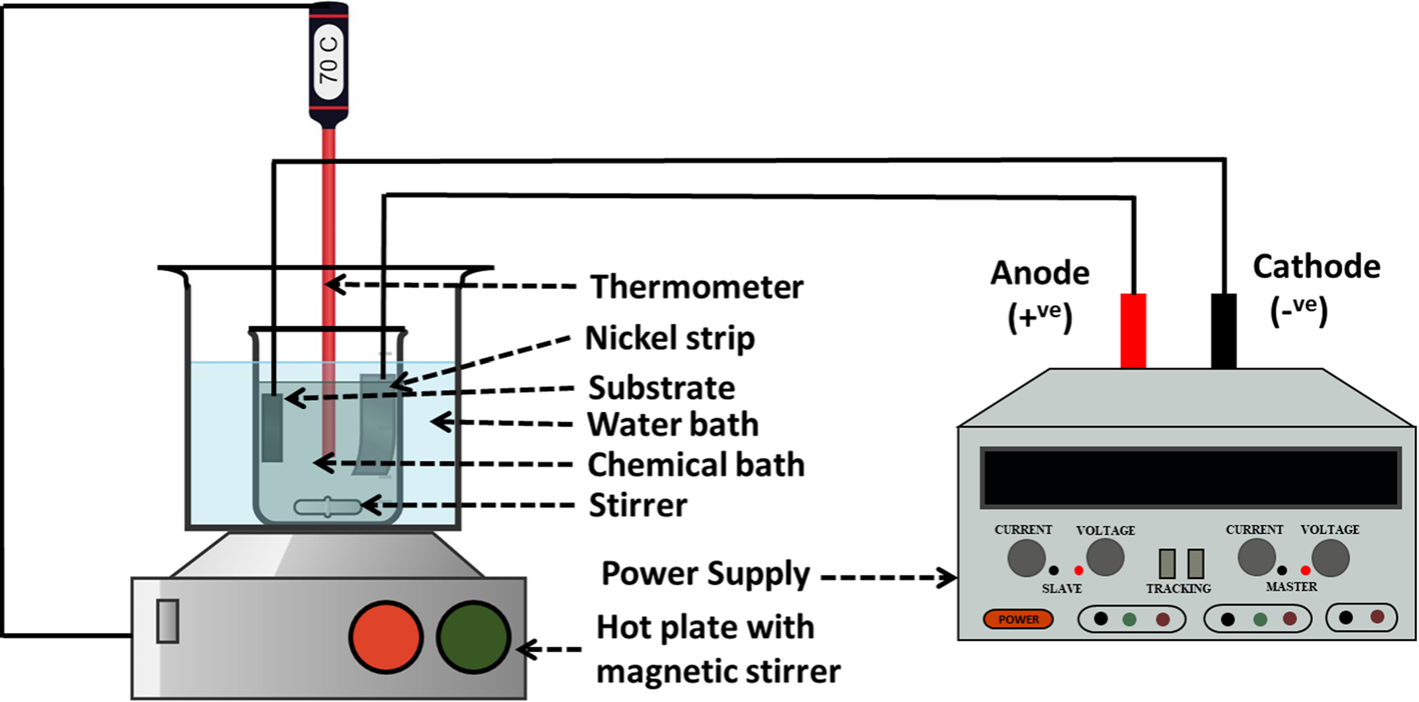
Schematic diagram of the electrodeposition process to develop Ni–P–TiC composite coatings.

The electrodeposition of Ni–P and Ni–P–TiC composite coatings was carried out on the mild steel substrate. Firstly, the mild steel sheet was cut down to the 32 mm square sheets through sheet metal operation. The mild steel samples were then polished to obtain a mirror-like surface with SiC abrasive papers of grit size 120, 220, 320, 500, 800, 1000, and 1200. The substrates were washed with soap and water before moving to the next abrasive paper. After grinding, the substrates were sonicated in the acetone for half an hour. One side of the substrates was covered with insulating tape to avoid electrodeposition on both sides of the substrates. The substrates were activated in 20% HCl solution for about 45 s, rinsed in distilled water, and finally put in the coating bath. During the electrodeposition process, the dc power supply’s negative electrode was connected to the substrate forming a cathode, and the positive electrode of the power supply was connected to the nickel sheet to provide an anode. The schematic diagram of the electrodeposition experimental setup is represented in Fig. 1. The nickel sheet (anode) and the substrate (cathode) were placed parallel and face to face each other at a distance of approximately 30 mm in the coating bath. The optimized electrodeposition conditions are tabulated in Table 1. Ni–P and Ni–P–TiC composite coatings were developed at 65 °C ± 2. The time of the coatings is half an hour from the start of the power supply. The coating bath was agitated at 300 ± 5 rpm for 60 min before initiating the electrodeposition process to avoid settling down of the TiC particles. The coating bath was kept agitated during the entire coating process at 300 rpm for uniform distribution of reinforcing particles into the Ni–P matrix.

**Table 1:** Optimized bath composition and parameters for co-electrodeposition of Ni–P–TiC composite coatings.

Chemical bath and operating conditions	Bath Ni–P/TiC
Nickel Sulfate hexahydrate	250 g L^−1^
Nickel Chloride hexahydrate	15 g L^−1^
Boric acid	30 g L^−1^
Sodium Chloride	15 g L^−1^
Phosphoric acid	6 g L^−1^
Sodium hypophosphite	20 g L^−1^
TiC particles concentration	0, 0.5 g L^−1^, 1 g L^−1^,1.5 g L^−1^ and 2 g L^−1^
pH	2.0 ± 0.2
Bath temperature	65 ± 2 °C
Deposition time	30 min
Current density	50 mA/cm^2^
Bath agitation	300 rpm

### Sample characterization

The thickness of the synthesized Ni–P and Ni–P–TiC composite coatings was determined by thickness gauge (model BDYSTD-E, USA). Structural characterization of the synthesized coatings was carried out employing X-ray diffractometer (PANalytical, Empyrean, UK) fitted with Cu Kα radiations with the scanning step of 0.02° in the range of 2θ from 10° to 90°. The field emission scanning electron microscope (FE-SEM-Nova Nano-450, Netherlands), atomic force microscopy (AFM-USA) and high-resolution transmission electron microscope (HR-TEM FEI : TECNAI G2 FEG 200 kV) were used to perform the morphological study. The composition of the prepared coatings was also determined by X-ray photoelectron spectroscopy—XPS (Kratos Analytical Ltd, UK) using a monochromatic Al-Kα X-Ray source. The hardness of the prepared coatings was tested with Vickers microhardness tester (FM-ARS9000, USA). The measurement of the microhardness was carried out at 100 gf with the dwell time of 10 s on the surface of the coatings. The nanoindentation measurements were performed employing AFM device MFP-3D Asylum research (USA) equipped with silicon probe (Al reflex coated Veeco model-OLTESPA, Olympus; spring constant: 2 Nm^−1^, resonant frequency: 70 kHz). All measurements were carried out under ambient conditions using standard topography A.C. air (tapping mode in the air). The indentation was performed with Berkovich diamond indenter tip with a maximum 1mN indentation force (loading and unloading rate: 200 µN/s and dwell time at maximum load: 5 s). Oliver and Pharr's method was used to find contact penetration from the unloading curves. The electrochemical impedance spectroscopy (EIS) studies were carried out with Gamry cell in which saturated silver/silver chloride (Ag/AgCl) was used as the reference electrode, whereas graphite and prepared coated samples were employed as counter and working electrodes, respectively. EIS was measured by AC signal with 10 mV of amplitude within the frequency range of 10^5^–10^−2^ Hz at open circuit potential. Moreover, potentiodynamic studies were carried out at ambient room temperature with a scan rate of 0.167 mV s^−1^ after the determination of open circuit potential for more than 10 min of stabilization of complete cell. A constant surface area of 0.765 cm^2^ of all tested samples was exposed to 3.5 wt% NaCl solution in the entire study^33,42,43^.

## Results and discussion

### XRD analysis

**Figure 2:**
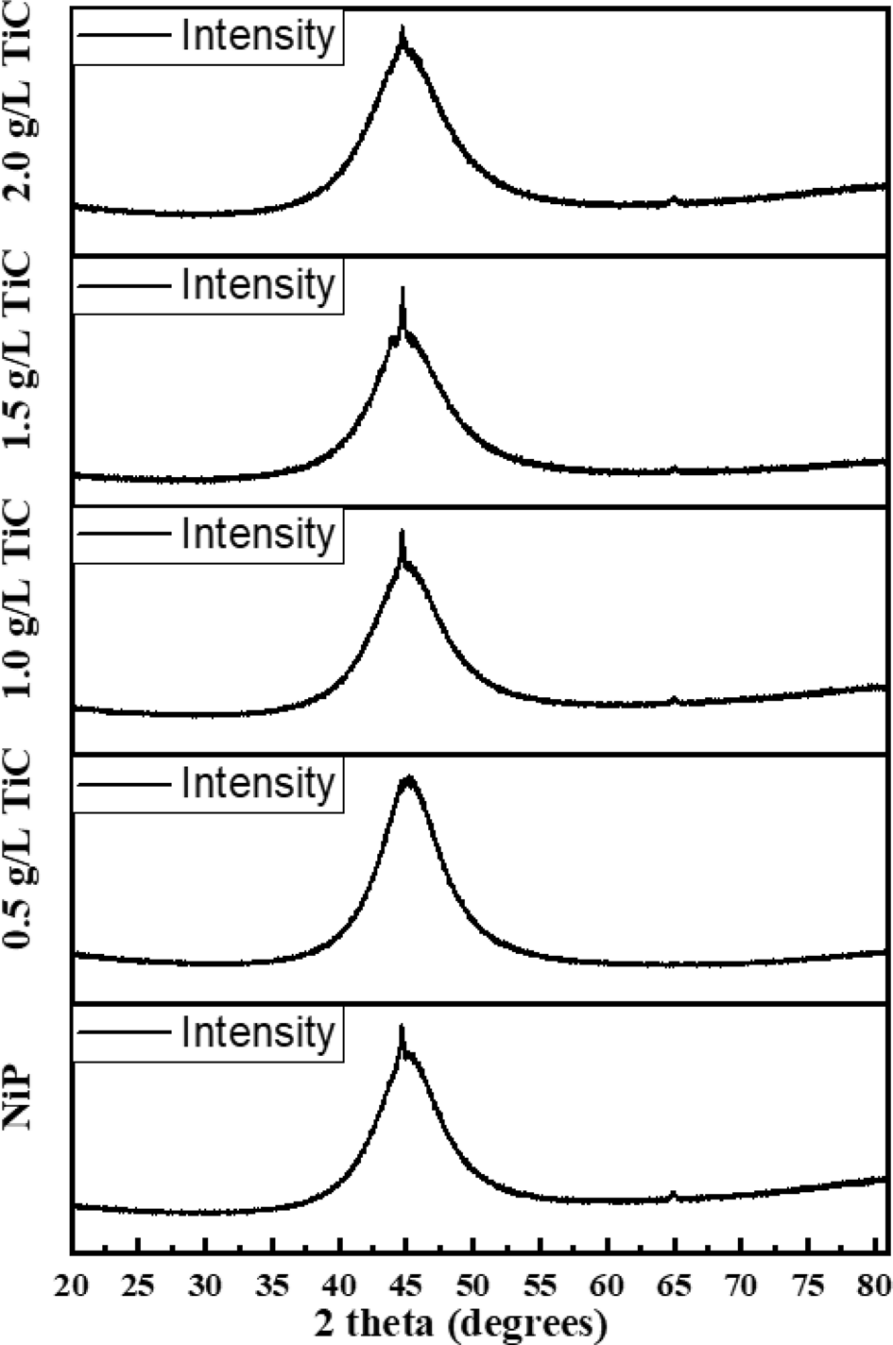
XRD spectra of Ni–P and Ni–P–TiC composite coatings containing various concentrations of TiC particles.

The structural analysis of the electrodeposited Ni–P and Ni–P–TiC composite coating was carried out through XRD and the spectra of NiP and Ni–P–TiC composite coatings containing various compositions of TiC (0, 0.5, 1.0, 1.5, 2 g L^−1^) are shown in Fig. 2. The semi-amorphous structure of the coatings can be deduced from the broad peaks in all the cases, and the broad peak located at 2Ɵ ~ 45.5 can be assigned to the Ni (111) plane of face-centered cubic (FCC) structure. The formation of an amorphous structure can be ascribed to the lattice distortion experienced by the nickel crystal structure due to the presence of phosphorous atoms, which hinders the propagation of face-centered cubic occupancy of nickel atoms^44^. The amorphous nature of the coatings has already been reported^10,15,45^ along with nanocrystalline structure as reported in the literature^46,47^. The diffraction peaks of the TiC were not observed in the XRD spectra, probably due to their low contents in the Ni–P matrix. Similar results have also been reported in the literature^29,48^.

### XPS analysis

**Figure 3:**
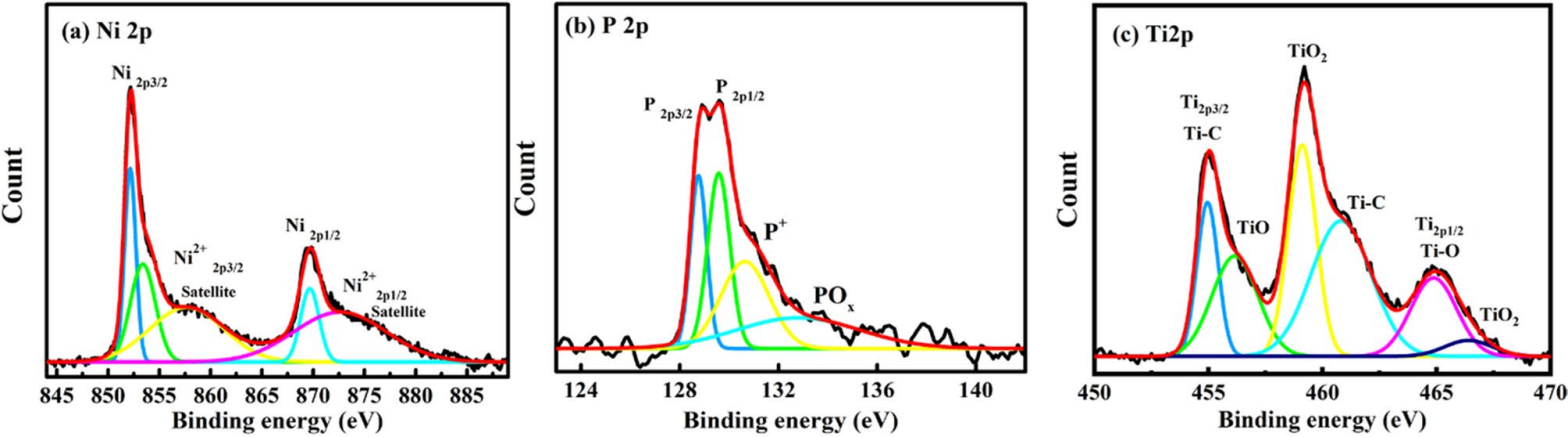
XPS spectra presenting the elemental composition of Ni–P/1.5 g L^−1^ TiC composite coatings, (**a**) Ni2p, (**b**) P2p and (**c**) Ti2p.

The presence of TiC in the Ni–P TiC composite coatings was confirmed using XPS analysis. To avoid any repetition, the fitted data of individual photoionizations and their corresponding chemical states only the 1.5 g L^−1^ TiC composition is presented in Fig. 3. High energy resolution spectra of Ni2p (Fig. 3a) region contains two distinct ionizations: Ni 2p3/2 and Ni 2p1/2 at 852.2 eV and 869.9 eV assigned to Ni in the metallic state, whereas the peaks of Ni^2+^ at 853.3 eV, 857.6 eV, and 872.7 eV corresponds, respectively to the NiO and/or Ni(OH)_2_ of Ni 2p3/2 and Ni 2p1/2. The high intensity peak for nickel proves the presence of metallic nickel. The formation of Ni(OH)_2_ and NiO can be linked to the presence of hydroxyl ion from the aqueous electrolytic bath and other surface oxidation phenomenon^33,49^. Concerning the P2p ionization, the peaks at 128.8 and 129.5 eV can be assigned to the elemental phosphorous (P) in the bulk of electrodeposited Ni–P–TiC composite coating, respectively (Fig. 3b). It can be noticed that the peak at 130.69 eV is due to (i) elemental phosphorus hypophosphite and/or (ii) intermediate phosphorous ions (P(I) and/or P(III)) valence which are presented in the inner portion of the protective film of the Ni–P coatings. However, peaks at 132.7 eV can be due to the combination of oxides and/or hydroxides (P_2_O_3_ and/or P-OH) chemical states^33^. The high-resolution spectra of the Ti2p spectrum were deconvoluted into three doublet peaks (Fig. 3c) of titanium carbide, based at 454.9 and 460.8 eV, titanium oxides at 456.1 and 464.8 eV and TiO_2_ at 459.2 and 466.4 eV as previously reported ^50,51^.

### Microstructural analysis

**Figure 4:**
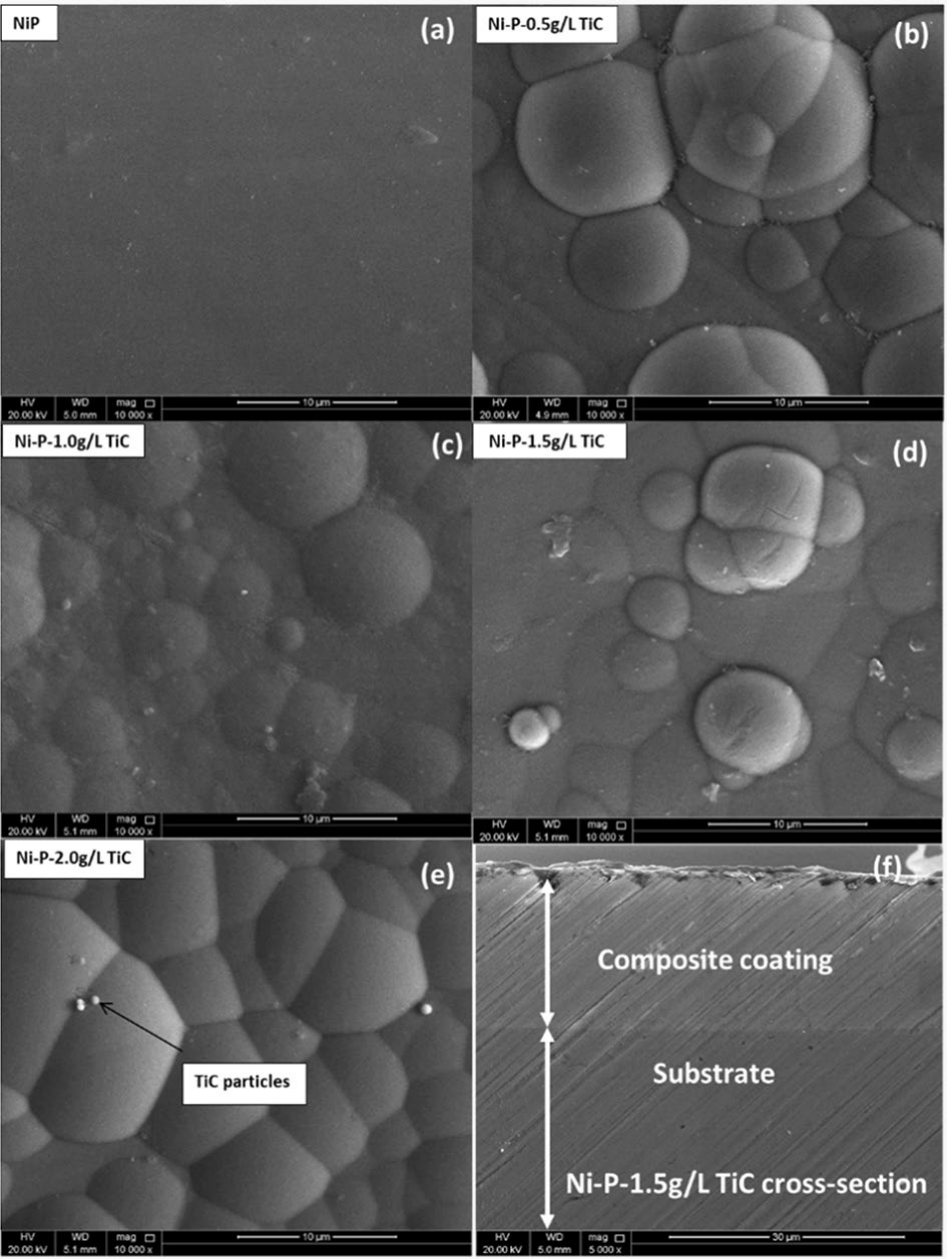
FE-SEM micrographs of the Ni–P (**a**) and Ni–P–TiC composite coating with various concentrations of TiC (**b**,**c**,**d**,**e**). A cross-sectional micrograph (**f**) of Ni–P–TiC composite coatings with 1.5 g L^−1^ of TiC.

The morphology of the Ni–P and Ni–P/TiC composite coatings containing various concentrations of TiC particles was studied with FE-SEM as specified in Fig. 4. Ni–P coatings (Fig. 4(a) does not show the formation of a well-defined nodular structure. A similar morphology of Ni–P coatings has been reported in the literature^29,52^. On the other hand, FE-SEM micrographs of Ni–P–TiC composite coatings (Fig. 4b–e) show the compact, nodular morphology without any noticeable defects. The presence of TiC particles can also be observed in the FE-SEM images, especially at the 2.0 g L^−1^ of composition in good agreement with literature^33,53^. Figure 4f shows the cross-section of Ni–P–TiC (1.5 g L^−1^) composite coatings. A smooth and well-adherent coating, without any apparent defects can be observed, together with an uniform interface. A uniform coating thickness of ~ 15 µm is achieved.

The coating thickness was also measured with the coating gauge meter and presented in Table 2. It can be noticed that the coating thickness under all identical conditions are similar, and there are no noticeable changes in the thickness. It is worthy of mentioning that the reported values are an average of five readings. A slight difference in thickness of coatings measured through FE-SEM analysis may be due to the surface preparation required for the test.

**Table 2:** Average thickness of Ni–P and Ni–P-TiC composite coatings measured with thickness gauge meter.

Coatings Composition	Average coating thickness
Ni–P	17 µm ± 2
Ni–P 0.5 g L^−1^ TiC	17 µm ± 2
Ni–P 1.0 g L^−1^ TiC	17.4 µm ± 2
Ni–P 1.5 g L^−1^ TiC	17.2 µm ± 2
Ni–P 2.0 g L^−1^ TiC	17.6 µm ± 2

**Figure 5:**
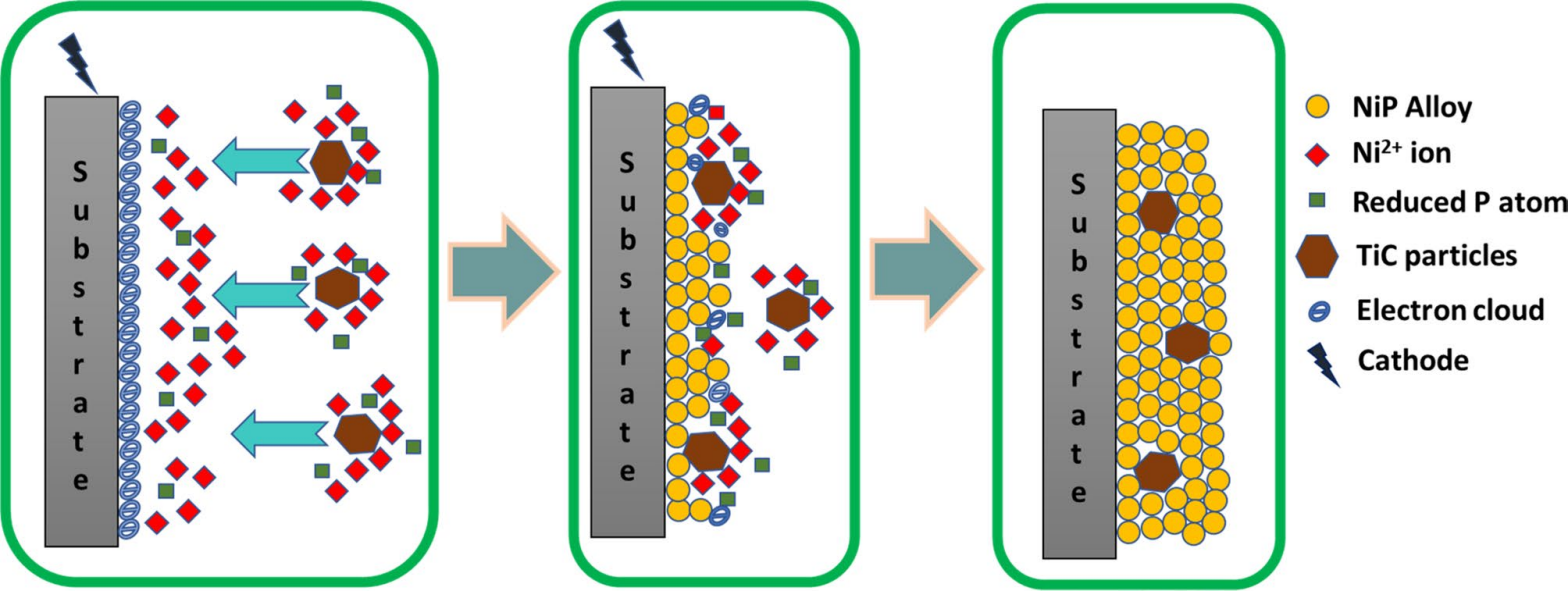
Schematic diagram for the co-deposition of TiC particles at the cathode (substrate) to form Ni–P–TiC composite coatings.

Co-deposition mechanism of various reinforcements in Ni–P matrix has been proposed by many researchers. Guglielmi^54^ proposed a model containing two steps in which firstly, particles adsorb weakly on the cathode surface by Van der Waals forces and then during the second stage strong adsorption by coulombic forces. This model fails to account for particle size and hydrodynamics of the deposition. Bercot et al.^55^ formulated a corrective factor to this model for accounting for magnetic stirring in their study, whereas Bahadormanesh and Dolati modified Guglielmi’s model for the deposition of a high-volume percentage of the second phase and carried out a parametric study^56^. Moreover, Fransaer et al. devised a trajectory model in which they presented an analysis of various forces on a spherical particle in a rotating disk electrode system^57^. According to Ceils et al.^58^, the electrodeposition mechanism may consist of five steps; (i), formation of an ionic cloud around the reinforcement particles, (ii) movement of reinforcement particles by forced convection towards the hydrodynamic layer of the cathode, (iii) diffusion of the particle through double layer, (iv) adsorption of the particle along with the ionic cloud at the cathode surface and (v) reduction of the ionic cloud leading to an irreversible entrapment of reinforcement particles in the metal matrix. As per the above discussion, it seems there are mainly three steps involved in the co-deposition of the reinforcement particles, such as TiC during the electrodeposition process; (i) movement of particles from bulk electrolyte to hydrodynamic boundary layer of the cathode which are governed by a combination of forced convection and electrophoresis, (ii) diffusion and adsorption of particles at the cathode due to Van der Waal forces, and (iii) permanent incorporation of particles due to the reduction of ionic cloud around the reinforced particle. This three-step phenomenon can be described in the schematic diagram in Fig. 5.

**Figure 6:**
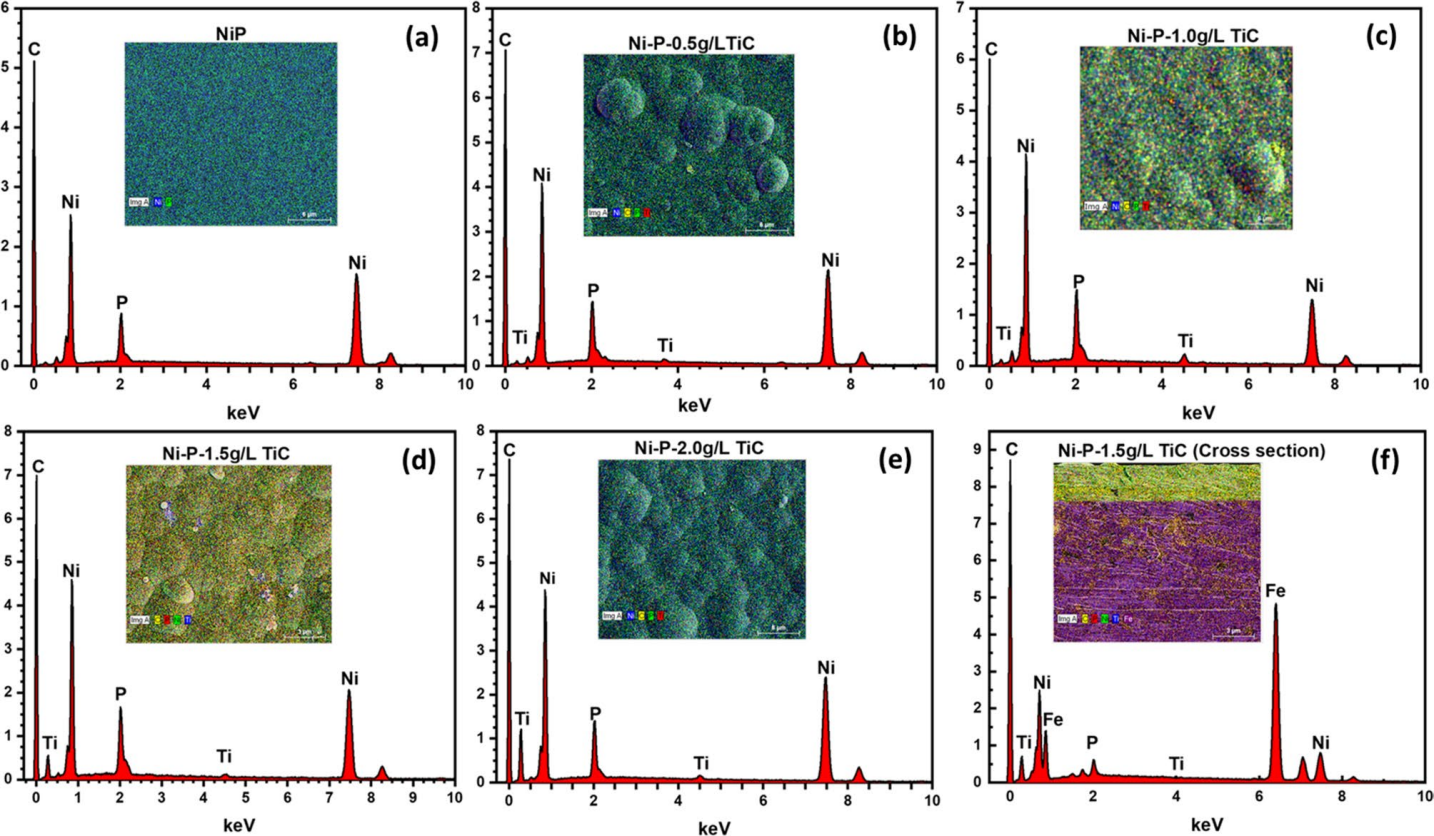
EDS analysis along with elemental mapping of Ni–P (**a**) and various compositions of Ni–P–TiC composite coatings, (**b**) 0.5 g L^−1^, (**c**) 1.0 g L^−1^, (**d**) 1.5 g L^−1^, (**e**)2.0 g L^−1^ and (**f**) cross-section of 1.5 g L^−1^ of Ni–P–TiC composite coatings.

The co-electrodeposited of TiC in the Ni–P matrix was further evaluated with EDS analysis. The EDS analysis of Ni–P and Ni–P-TiC composite coatings containing various concentrations of TiC particles, is presented in Fig. 6a–f. The elemental mapping of Ni–P /TiC composite coatings is shown as an inset of Fig. 6. The presence of titanium (Ti), carbon, (C), Phosphorus (P), and nickel (Ni) confirm the incorporation of TiC particles into the Ni–P matrix. Table 3 shows the weight percentage of various elements in the as prepared composite coatings. As for Ni–P coating, nickel constitutes almost 89.51 wt.% and the remaining is balanced by phosphorus. Introduction and increase of the concentration of TiC powder in the chemical bath does affect the concentration of nickel in the deposit, which appreciably decreases without significant effect over the phosphorus content which remains around 10 wt.% in all the coatings. The titanium content in the deposits increases from 0.39 to 0.84 wt.% when the concentration in the chemical bath is increased from 0.5 to 2.0 g L^−1^. However, the excessive weight percentage of carbon can be attributed to the combination of various effects such as presence of carbon in the titanium carbide compound, impurities related to environment and surface preparation for the microscopic analysis. Incorporation of TiC particles can be inferred from the titanium peaks in the EDS plot of 0.5, 1.0, 1.5, 2.0 g L^−1^ and cross-section of 1.5 g L^−1^ of TiC. The carbon peak in all the plots can be attributed to the steel substrate's carbon composition due to background interference as previously reported by Pouladi et al.^59^. Peaks of iron are also observed in the cross-sectional EDS analysis which can be ascribed to the steel substrate. Further, corresponding EDS elemental mapping results shown as an inset of corresponding compositions depicts the clear distribution of Ni, P, and TiC particles in the Ni–P matrix.

**Table 3:** EDS quantitative analysis of Ni–P and Ni–P-TiC composite coatings.

S. no	Sample designation	Ni (wt.%)	P (wt.%)	Ti (wt.%)	C (wt.%)
1	Ni–P	89.51	10.49	–	–
2	Ni–P-0.5 g L^−1^ TiC	73.47	9.94	0.39	16.2
3	Ni–P-1.0 g L^−1^ TiC	69.74	9.82	0.64	19.8
4	Ni–P-1.5 g L^−1^ TiC	66.19	10.52	0.79	22.5
5	Ni–P-2.0 g L^−1^ TiC	66.58	9.68	0.84	22.9

**Figure 7:**
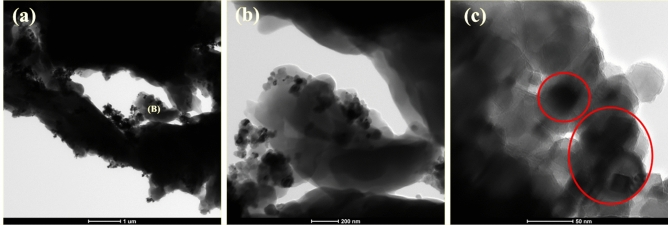
TEM micrographs of Ni–P-2.0 g L^−1^ TiC at various magnification of (**a**) high magnification (**b**) magnified portion marked (B) in (**a**) and (**c**) showing an interface of the Ni–P matrix and TiC reinforcement.

In order to further investigate the microsctructural properties of the deposit, high resolution-transmission electron microscopy analysis were carried out for the Ni–P-2.0 g L^−1^ TiC. Figure 7 shows the TEM bright field micrographs of electrodeposited Ni–P-2.0 g L^−1^ TiC composite coating at various magnifications. All the images clearly reveal the presence of a separate second phase of TiC particles within the Ni–P matrix. Figure 7a presents a low magnification micrograph of the composite coating. The excessive darkness is due to the thickness of the coating deposited on the copper grit for TEM analysis. Figure 7b is the enlarged image at the marked location (B) in Fig. 7a presenting the amorphous structure of the composite coating with the lighter region corresponding to the nickel lattice formation as also reported by Huang et al. in their exhaustive study of microstructure in the Ni–P coating^60^. An irregular dark network is observed in the Fig. 7b which is prevalent to the mid-high phosphorus content within the electrodeposited composite coatings as previously reported^60,61^. Figure 7c is the micrograph at very high magnification presenting the cubical polygonal structure of the reinforced titanium carbide embedded in the Ni–P matrix. The matrix-reinforcement interface can be clearly distinguished as comparatively sharp contrast can be identified in the micrographs. According to literature, titanium carbide particles are reported to have regular polygonal cubical structure^62^.

**Figure 8:**
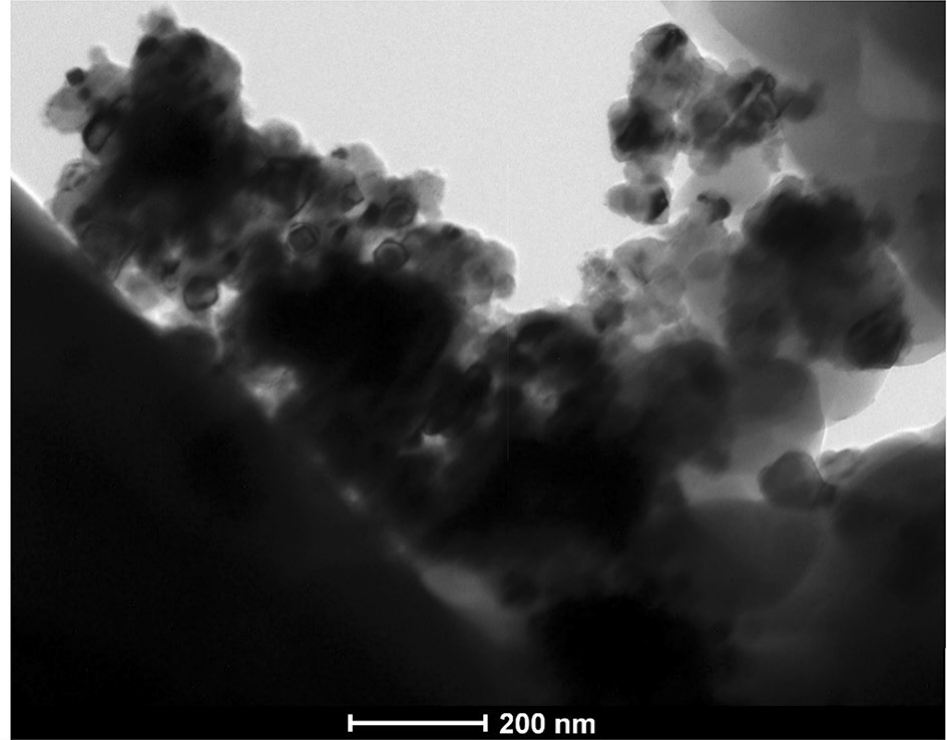
TEM micrograph of Ni–P-2.0 g L^−1^ TiC presenting the agglomeration of the particles in the Ni–P matrix.

FE-SEM images could not accurately provide the evidence of aggregation or agglomeration of TiC particles during the fabrication of the Ni–P-2.0 g L^−1^ TiC composite coating. TEM analysis further confirms the agglomeration or aggregation of the cubical polygonal TiC particles, which are visible in Fig. 8 for the Ni–P-2.0 g L^−1^ TiC. Agglomeration of the particles in composite coatings has been confirmed through TEM micrograph as reported in literature^61,63^.

**Figure 9:**
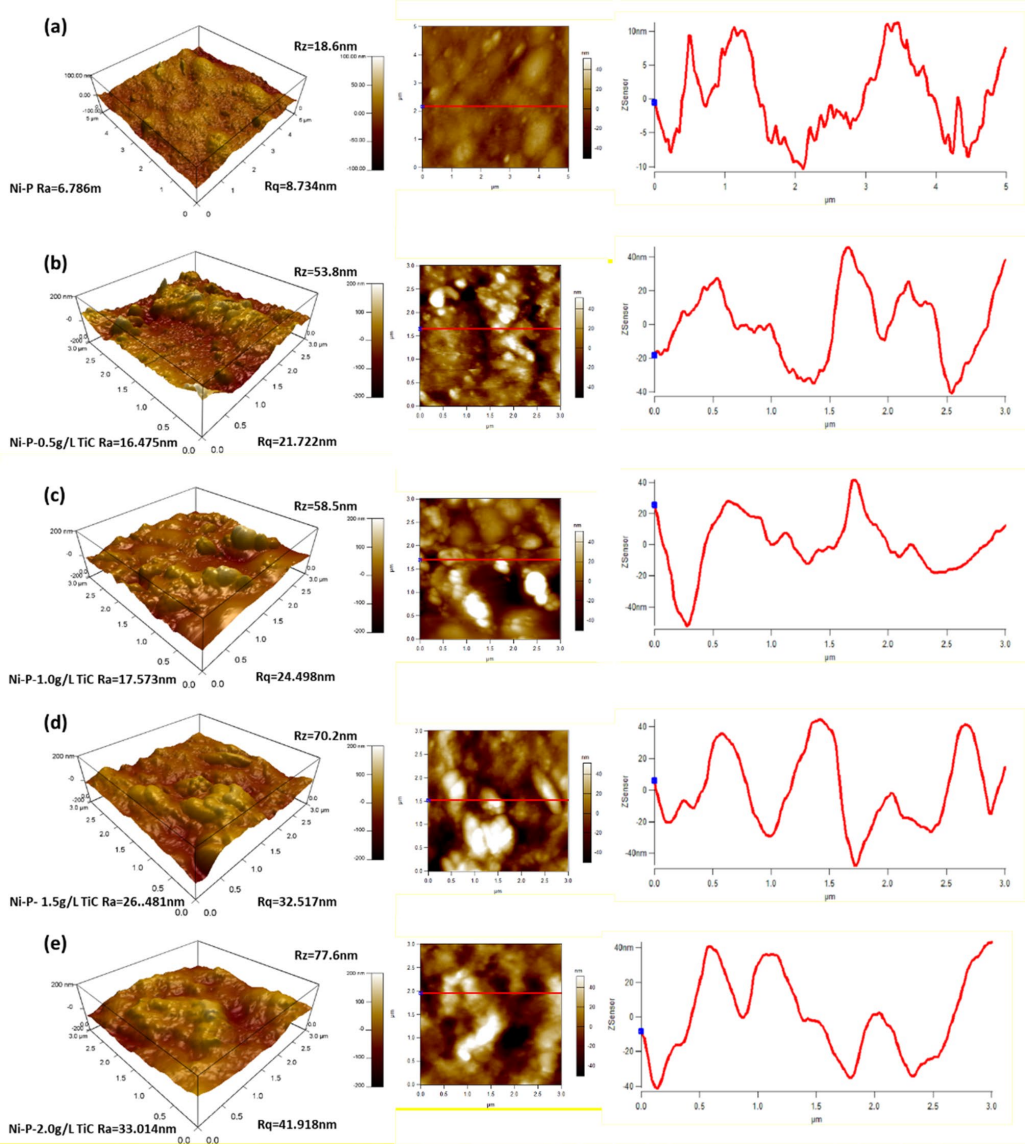
3D-AFM micrograph along with their corresponding surface roughness profiles of the (**a**) Ni–P, Ni–P–TiC composite coatings (**b**) 0.5 g L^−1^, (**c**) 1.0 g L^−1^, (**d**) 1.5 g L^−1^, and (**e**) 2.0 g L^−1^ of TiC particles.

The surface topography of the electrodeposited Ni–P and Ni–P–TiC composite coatings was investigated through atomic force microscopy (AFM). Three-dimensional images of Ni–P and Ni–P/TiC composite coatings with the various compositions of TiC particles are presented in Fig. 9a–e. It is observed that the Ni–P coatings indicate a relatively smooth surface when compared with the Ni–P–TiC composite coatings. The Ni–P–TiC composite coatings' surface is composed of valleys and intrusions due presence of TiC particles into the Ni–P matrix that provides a rougher texture. The quantitative analysis of surface topography indicates that the addition of TiC particles into the Ni–P matrix has resulted in an increase in the surface roughness. The average surface roughness (Ra) increases with the increasing amount of TiC particles and the average value increased from 6.786 nm (Ni–P coatings) to 33.014 nm (Ni–P/TiC-2.0 g L^−1^), contributing five times enhancement in the surface roughness. Moreover, Rq (root mean square value of the roughness) is also presented which shows the similar trend as the average roughness as presented in the Fig. 9. Furthermore, Rz values also displays the similar increasing trend from 18.6 nm roughness of Ni–P coating to the successive increase upto 53.8 nm, 58.5 nm, 70.2 nm and 77.6 nm for the increase in the concentration of TiC particles of 0.5 g L^−1^, 1.0 g L^−1^, 1.5 g L^−1^ and 2.0 g L^−1^ in the chemical bath. The increase in the surface roughness with an increasing amount of TiC particles can be attributed to the presence of insoluble and hard ceramic particles, which provides jerks and barriers to the free movement of the AFM cantilever tip. These findings are consistent with the previous studies^29,33^.

### Mechanical properties

#### Vickers microhardness

**Figure 10:**
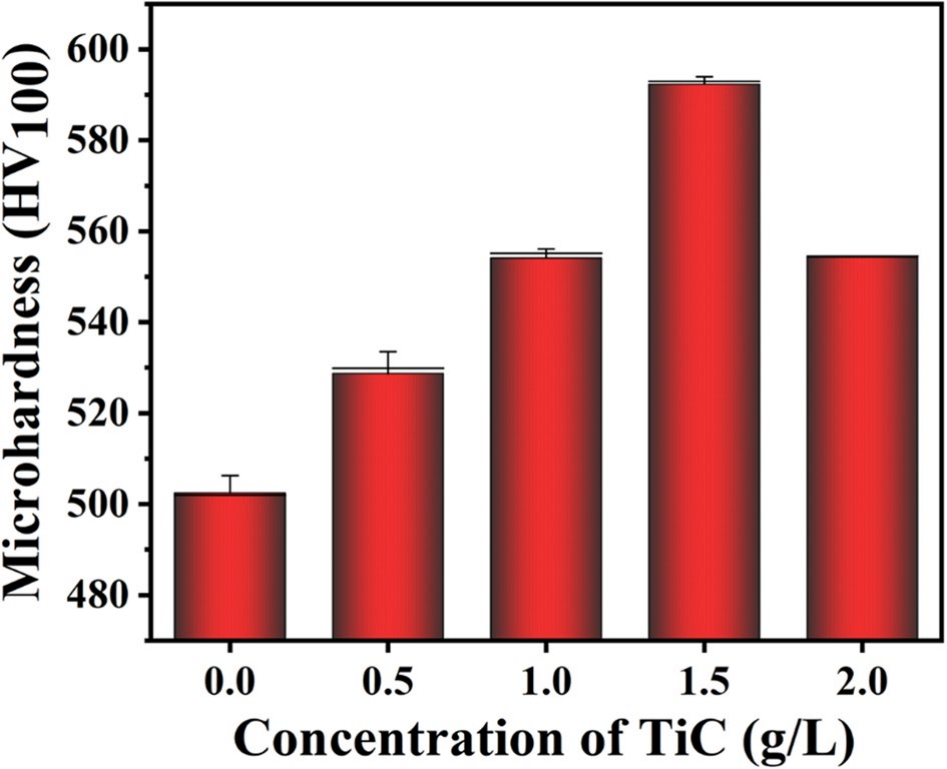
Vickers microhardness of Ni–P and Ni–P–TiC composite coatings containing various concentrations of TiC particles.

Vickers microhardness results of Ni–P and Ni–P–TiC composite coatings are presented in Fig. 10. As seen, Ni–P coating's hardness value is around 500HV, which increases to ~ 530 HV and ~ 550 HV on the incorporation of 0.5 g L^−1^ and 1 g L^−1^ of the TiC particles, respectively. The hardness value reaches its maximum value of ~ 593 HV at the composition of 1.5 g L^−1^. The increase in the hardness is about 19%, which can be attributed to the dispersion hardening effect and improvement in the load-bearing characteristics of the matrix due to the formation of a composite structure, aligned to previously reported literature^64,65^. After reaching to its terminal value, the microhardness decreases with further increase in TiC particles and it decreases to ~ 550 HV at 2.0 g L^−1^. A decrease in the hardness value at 2.0 g L^−1^ can be attributed to the excessive aggregation of the TiC particles in Ni–P matrix, which impairs the load-bearing properties of the Ni–P/TiC composite coatings. This observation is also consistent with previous reports^66^.

### Nanoindentation

**Figure 11:**
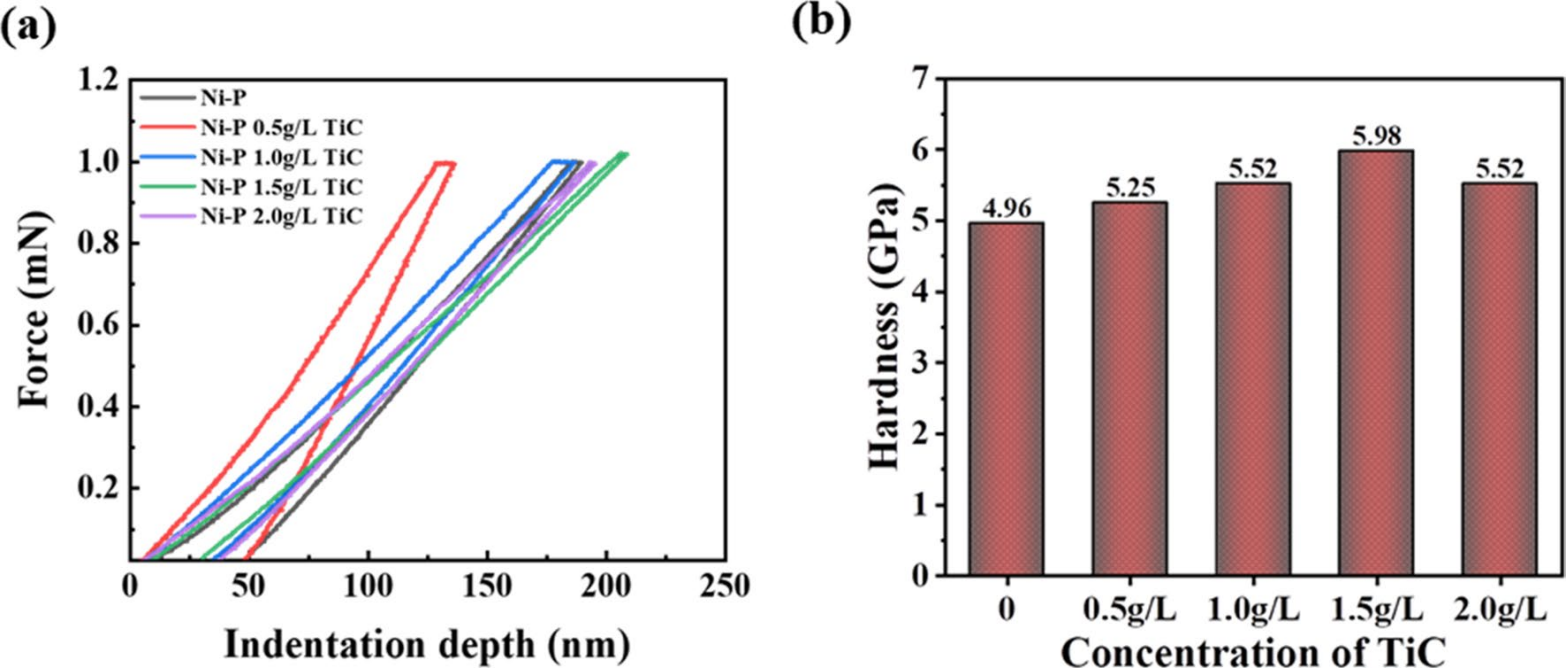
Nanoindentation results of Ni–P and Ni–P–TiC composite coatings containing various concentrations of TiC particles; (**a**) loading/unloading profiles and (**b**) hardness.

The indentation tests of the Ni–P and Ni–P–TiC composite coatings were performed to have an insight of the mechanical response of the developed coatings. The loading/unloading indentation profiles of Ni–P and Ni–P–TiC composite coatings containing various concentrations of TiC particles are presented in Fig. 11. A gradual decrease in indentation depth with an increasing amount of TiC particles in the Ni–P matrix is evident in Fig. 11a. The Ni–P coatings demonstrate an indentation depth of ~ 50 nm, which reduces to 23.67 nm at the composition of 1.5 g L^−1^ of TiC. The decrease in depth is due to the enhancement in the hardness of the coatings, which is directly associated with the dispersion hardening effect and improvement in the load-bearing properties, as explained previously. It can be further noticed that there is a decrease in the indentation depth of ~ 7 nm at the terminal composition (2.0 g L^−1^ TiC). This is because of the fact that an excessive amount of reinforcement accumulates in the matrix and thus harms the mechanical properties are in agreement with previous studies^67,68^. The maximum decrease in the indentation depth is observed at 1.5 g L^−1^ of TiC due to the uniform distribution of the reinforcing phase in the matrix without any significant agglomeration. The loading/unloading curves are uniform without any kinks, suggesting that the synthesized coatings are free of cracks and pores. For more accurate comparison, a quantitative analysis of the indentation results obtained through Oliver and Pharr technique is also represented in Fig. 11b. It can be noticed that the hardness of Ni–P coatings is 4.96 GPa, which increases with increasing concentration of TiC particles in the Ni–P matrix, reaching to its terminal value of 5.98 GPa at the composition of 1.5 g L^−1^. Further increase of TiC particles concentration in the Ni–P matrix decreases hardness and it attains a value of 5.52 GPa at the TiC composition of 2.0 g L^−1^. This result further supports the observation that incorporation of ceramic TiC increases the hardness of the NiP matrix, in good agreement with literature^33,69^. The decrease in the hardness for 2.0 g L^−1^ can be due to agglomeration of TiC particles in the Ni–P matrix. The nanoindentation results are in agreement with the Vickers microhardness test results.

## Corrosion behavior

### Electrochemical impedance spectroscopy (EIS)

**Figure 12:**
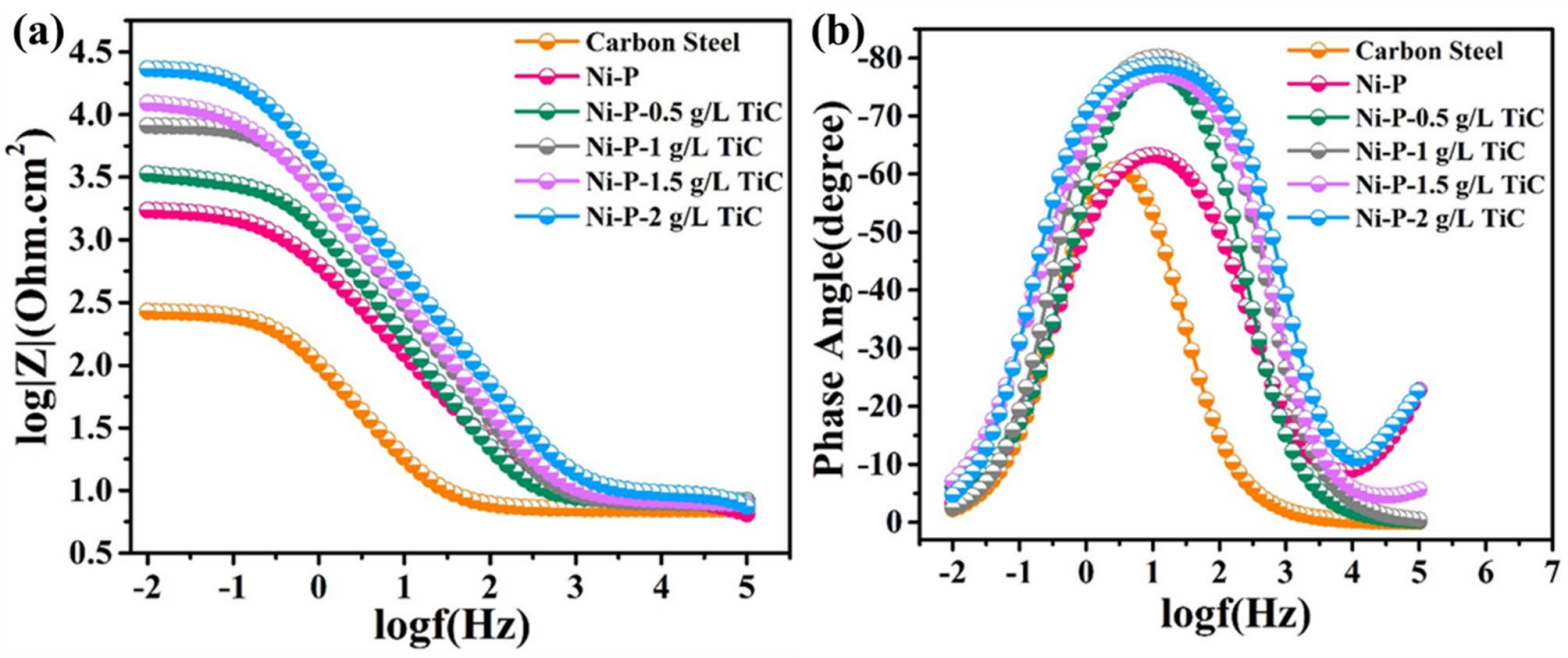
(**a**) Bode plots of the substrate, Ni–P, and Ni–P–TiC composite coatings containing the magnitude plot and (**b**) phase angle plot after 2 h of immersion in 3.5wt% NaCl solution.

**Figure 13:**
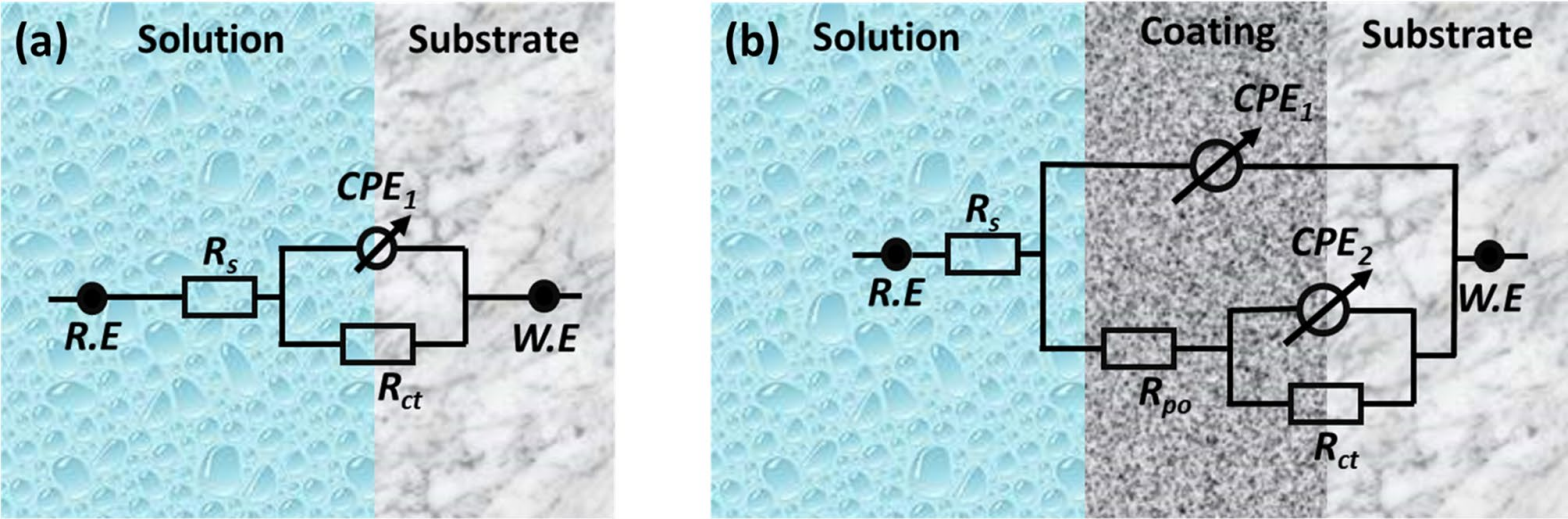
Equivalent electric circuit used for fitting the experimental EIS data for (**a**) polished carbon steel used as substrate, (**b**) Ni–P and Ni–P–TiC composite coatings containing different concentrations of TiC particles.

The corrosion resistance of the coatings was studied through electrochemical impedance spectroscopy (EIS) and potentiodynamic polarization techniques. The EIS plots (Bode plots) of the substrate (carbon steel), NiP, and NiP-TiC composite coatings containing various concentrations of TiC are presented in Fig. 12a,b. Experimental data were fitted using an equivalent circuit based on a modified Randle circuit. It is composed of two-time constants in cascade assigned to the composite coatings and metal-coating interface exposed at the bottom of conductive paths, as presented in Fig. 13a,b. The various elements in the circuit account for: Rs—electrolyte resistance, Rpo—pore resistance, Rct—polarization resistance, and constant phase elements (CPE1 and CPE2) instead of capacitors to account for surface inhomogeneity. The constant phase elements can be calculated by the following equation^33^:

$$\frac{1}{ZCPE}={Q\left(j\omega \right)}^{n}$$

where Q is the admittance and ω is the angular frequency of the alternating signal and n is the exponent of CPE which determines the capacitance nature, i.e., when “n” approaches unity, the CPE approaches to pure capacitance and the element behaves like an ideal capacitor^33^.

Referring to Fig. 12, the medium–high-frequency regions of the Bode plot for carbon steel evidence one time constant, while for the coated samples there is a broadening of the phase angle, suggesting two overlapped time constants—the one associated to the composite coating and another to the interfacial phenomena at the bottom of pores formed in the coating. The magnitude plot indicates that the corrosion resistance of the carbon steel sample is very low ~ 270 Ω cm^2^, a value that was obtained after fitting the experimental data using the proposed equivalent circuit (Fig. 13a). Ni–P coatings show an improvement in the impedance value of one order of magnitude which can be ascribed to the formation of the hypophosphite layer due to electrochemical reactions of the salt solution with the surface of Ni–P coating^70,71^. The inclusion of secondary phase TiC particles in the Ni–P matrix further changes the impedance response, leading to the broadening of the phase angle plot. This trend indicates, by the one hand, a more protective composite coating (shift towards higher frequencies) and, on the other hand, the presence of other processes (decreased corrosion activity) as previously reported in literature^33,42^. The increased impedance in the composite coatings can be attributed to the reduction on the number active corrosion sites due to the occupancy of inert and corrosion-resistant TiC particles. The Ni–P-0.5 g L^−1^ TiC showed almost doubled impedance values compared to a simple Ni–P coated sample (Fig. 12). An increase in the concentration of TiC particles from 0.5 g L^−1^ up to 2.0 g L^−1^ has successively increased the corrosion resistance and the maximum impedance values for Ni–P-2.0 g L^−1^ TiC reaches 23 kΩ cm^2^ showing an improvement of ~ 92% when compared to Ni–P coatings. An increase in the pore resistance can be due to the presence of TiC particles in the pores of Ni–P matrix that decreases the number of conductive paths and increases the surface roughness as observed in AFM results^49^. Improvement in the polarization resistance can be related to the successive increase in the reinforcement of TiC particles in the Ni–P matrix which hinders the electrolyte from reaching the substrate, decreasing the number of active sites and hence providing additional protection against corrosion^33,42,49^.

**Figure 14:**
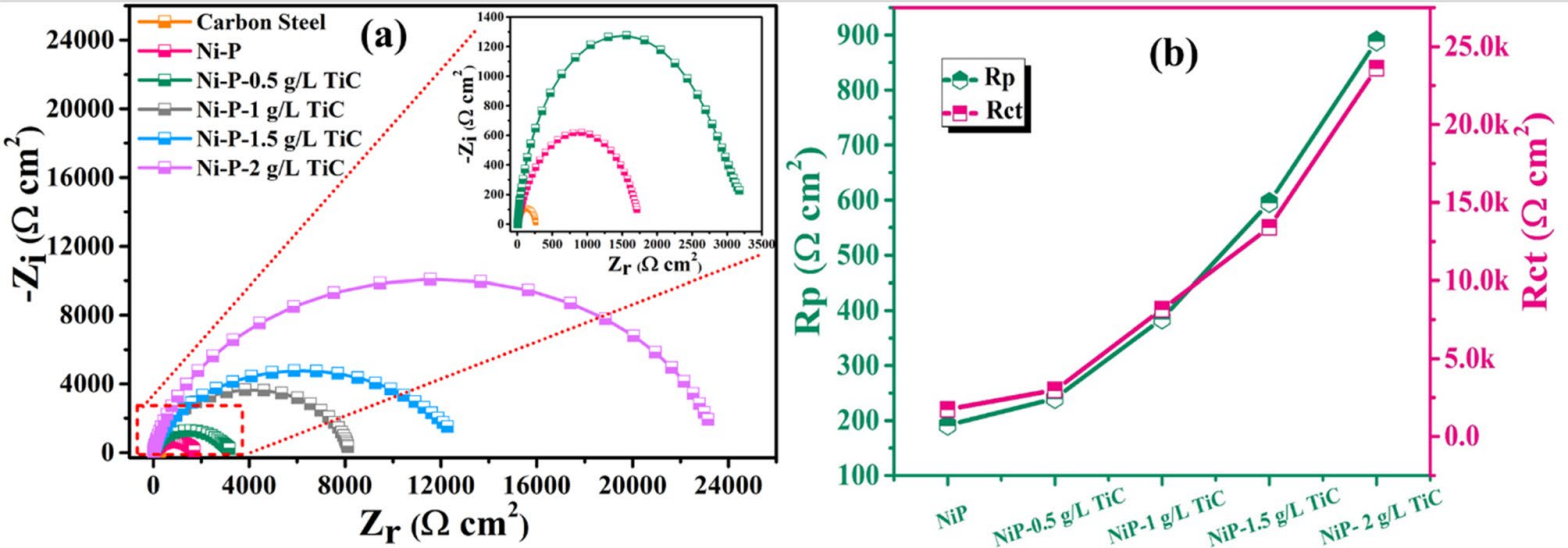
(**a**) Nyquist plots for carbon steel (substrate) and Ni–P–TiC composite coatings along with fitted resistance values vs. the concentration of TiC particles after the 2 h of immersion in 3.5wt% NaCl solution (**b**) evolution of R_po_ and R_ct_ with the TiC particles concentration.

Figure 14a depicts the Nyquist plots for carbon steel (substrate), Ni–P and Ni–P–TiC composite coatings containing various concentrations of TiC particles. Nyquist plots of Ni–P coatings and Ni–P–TiC composite coatings demonstrate distinct capacitive loops. The experimental plots for the coated samples were fitted using the two-time constant equivalent electric circuit described in Fig. 13b and the fitting goodness is represented in Fig. 14 in the Nyquist plots. The capacitive loop diameter evidences a successive increase, confirming the higher corrosion resistance in the presence of TiC particles. Figure 14 depicts the evolution of the pore resistance and polarization resistance over time. The incorporation of TiC particles in the Ni–P matrix increases the pore resistance in the coating and acts as a barrier by that delays electrolyte uptake. The decrease of the active surface area is responsible for the increase in the polarization resistance (Rct) as shown in Fig. 14b. Moreover, increasing the concentration of TiC particles in the chemical bath leads to a decrease in the active region and, therefore, increases the corrosion resistance of the composite coatings. The enhancement in the corrosion resistance of the NiP coating in the presence of various concentrations of TiC can be enumerated by the combined effect of (i) Inert TiC particles reduce the active area in the NiP alloy (ii) TiC particles are assumed to block the pores by filling them and restricting the diffusion of the Cl^−^ ions towards the metal surface and (iii) double-layer capacitance reduces. These findings are consistent with the previous studies^9,33,42,49^.

### Potentiodynamic polarization analysis

**Figure 15:**
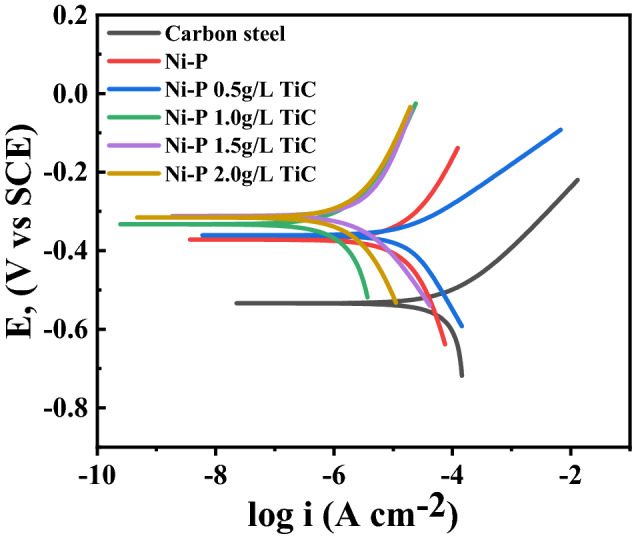
Potentiodynamic profiles of carbon steel, Ni–P and Ni–P–TiC composite coating with increasing concentration of TiC.

The corrosion resistance of the carbon steel, Ni–P, and Ni–P–TiC composite coatings containing various concentrations of TiC particles was also studied by d.c. potentiodynamic polarization employing a scan rate of 0.167 mV s^−1^ as shown in Fig. 15. Electrochemical parameters such as corrosion potential (Ecorr), corrosion current density (Icorr), anodic Tafel slope (βa), and cathodic Tafel slope (βc) were extrapolated from the fitted curve and tabulated in Table 4. Moreover, the corrosion protection efficiency (PE %) was calculated from the formula as reported^33^.

**Table 4:** Electrochemical parameters derived from the potentiodynamic polarization curve of carbon steel, Ni–P, and Ni–P-TiC composite coating containing various concentration of TiC particles.

Composition	βa (V/decade)	βc (V/decade)	Icorr (µA cm^−2^)	Ecorr (mV)	PE%
Carbon steel	0.09617	0.2275	55.94	− 534.0	
Ni–P	0.3514	0.6088	38.43	− 372.0	31.3%
Ni–P 0.5 g L^−1^ TiC	0.1059	0.2664	25.62	− 361.0	54.2%
Ni–P 1.0 g L^−1^ TiC	0.4342	0.2902	7.79	− 333.0	86.0%
Ni–P 1.5 g L^−1^ TiC	0.4354	0.2434	6.49	− 312.0	88.4%
Ni–P 2.0 g L^−1^ TiC	0.384	0.4246	4.91	− 315.0	91.2%

PE% = 1-$\frac{{i}_{2}}{{i}_{1}}$ where i_1_ is the current density of the carbon steel and i_2_ is the current density of coated samples. The maximum value of current density (55.94 µA cm^−2^) is observed for carbon steel at a corrosion potential of -533 mV, the most cathodic one observed in Fig. 15. The current density decreases to 38.43 µA cm^−2^ for the Ni–P coatings and further decreases with increasing concentrations of TiC particles in the Ni–P matrix. Thus, the values of current density decrease to 25.62 µA cm^−2^, 7.79  µA cm^−2^, 6.49 µA cm^−2^ and 4.91 µA cm^−2^ for the 0.5 g L^−1^, 1.0 g L^−1^, 1.5 g L^−1^, and 2.0 g L^−1^ TiC composite coatings respectively. Moreover, the corrosion potential, becomes slightly more anodic for the Ni–P coatings and increases from ~ − 372 mV to ~ − 312 mV with increasing concentrations of TiC suggesting a slight inhibition of the anodic activity in the presence of the TiC particles in the Ni–P matrix. Interestingly, for the TiC concentrations of 1.0, 1.5 and 2.0 g L^−1^, the anodic current density is independent of the content of TiC particles, and significantly lower compared to the Ni–P coating. This trend evidences that the anodic activity is reduced in the presence of the TiC particles (for the 3 highest concentrations). However, the cathodic current density tends to increase as the concentration of particles increases, approaching the values observed for the Ni–P coating and steel. This indicates that the cathodic processes, mainly oxygen reduction, are favored by the presence of TiC particles. The potentiodynamic polarization results show that Ni–P coatings had lower corrosion resistance compared to steel, displaying a corrosion protection efficiency of ~ 31%. In such composite coatings, corrosion often initiates at grain boundaries of the nodules as result of the adsorption of chloride ions. The anodic activity leads to the formation of soluble NiCl_2_ which can proceed to formation of pits^72^. The corrosion protection efficiency, consequence of the decreased corrosion current density, increases with the increasing concentration of TiC particles in the Ni–P matrix. The highest corrosion protection efficiency (~ 90%) was achieved at a TiC concentration of 2.0 g L^−1^. To conclude, the inclusion of TiC particles in the Ni–P alloy matrix has improved the corrosion resistance as the concentration of TiC particles. By the one hand, the presence of particles inhibits the anodic reactions and, on the other hand, it contributes to reduce the number of active sites for the adsorption of chloride ions on the surface defects such as cracks and pores. Enhancement in the corrosion resistance by increased concentration of reinforcement is in good agreement with literature^33,35,36^.

## Conclusions

Ni–P–TiC composite coatings containing various concentrations of TiC particles were synthesized using the electrodeposition technique. The amount of TiC particles in the Ni–P matrix has a significant influence on its morphological, structural, mechanical, and corrosion protection properties. The hardness of Ni–P-TiC composite coatings increases with an increasing amount of TiC particles in the Ni–P matrix. However, an excessive amount of TiC particles (2.0 g L^−1^) leads to particles agglomeration and thus reduction in hardness. Electrochemical studies confirm the increased the corrosion protection offered by the Ni–P coatings with an increasing amount of TiC particles. The Ni–P–TiC composite coatings demonstrate superior mechanical and corrosion protection properties when compared to Ni–P coatings suggesting their utilization in many industries such as automobile, marine, electronic, oil, and gas industries.
